# Reference range: Which statistical intervals to use?

**DOI:** 10.1177/0962280220961793

**Published:** 2020-10-14

**Authors:** Wei Liu, Frank Bretz, Mario Cortina-Borja

**Affiliations:** 1 Mathematical Sciences & Southampton Statistical Sciences Research Institute, University of Southampton, Southampton, UK; 2 Novartis Pharma AG, Basel, Switzerland; 3 Department of Population, Policy and Practice Research and Teaching, Great Ormond Street Institute of Child Health, University College London, London, UK

**Keywords:** Nonparametric prediction interval, nonparametric tolerance interval, prediction interval, reference range, tolerance interval

## Abstract

Reference ranges, which are data-based intervals aiming to contain a pre-specified large proportion of the population values, are powerful tools to analyse observations in clinical laboratories. Their main point is to classify any future observations from the population which fall outside them as atypical and thus may warrant further investigation. As a reference range is constructed from a random sample from the population, the event ‘a reference range contains (100 P)% of the population’ is also random. Hence, all we can hope for is that such event has a large occurrence probability. In this paper we argue that some intervals, including the *P* prediction interval, are not suitable as reference ranges since there is a substantial probability that these intervals contain less than (100 P)% of the population, especially when the sample size is large. In contrast, a (P,γ) tolerance interval is designed to contain (100 P)% of the population with a pre-specified large confidence *γ* so it is eminently adequate as a reference range. An example based on real data illustrates the paper’s key points.

## 1 Introduction

The ‘Choose Wisely’ campaign was developed in the United States in 2012 by the American Board of Internal Medicine Foundation and was launched in the United Kingdom in 2016 by the Academy of Medical Royal Colleges. It aims to encourage a dialogue between clinicians and patients regarding the risk and benefits of interventions, and the practice of evidence-based treatment regimens.^1^ As described recently,^2^ this conversation often refers to the patient’s observed values of relevant clinical markers. Since the clinical laboratory provides comparator intervals to assist the clinician in determining a context for an individual value, a natural question from the patient is ‘Are my test results typical with respect to a healthy population?’. Although such assessment values are often referred to as the test’s normal range, this terminology should be discouraged as it implies that such a result has a binary ‘normal or abnormal’ quality which may lead to an arbitrary dichotomous interpretation of the patient’s health status.^2^ Instead, the terms ‘reference limits’ or ‘reference range’ should be used in this context.

Reference ranges are powerful tools in laboratory medicine to aid decision making^3^ and their use has become increasingly prevalent in clinical practice. Searching in the Web of Science engine at the time of writing for articles published between 1999 and 2019 with ‘reference range’ as a topic, we found 5431 articles of which 469 appeared in 2019, in contrast to 268 articles that appeared in 2009. These articles have collectively been cited by 91,034 publications of which 11,270 appeared in 2019, 2.4 times more than the number of citing publications 10 years earlier.

Apart from the important individual overtones for patients, incorrectly estimating the reference range of a sensitive clinical marker of physiological function has enormous public health implications. For example, underestimating the upper limit of a reference range would mean classifying a large number of people as diseased, thus affecting the doses of medication prescribed.^4^ Construction of appropriate reference ranges is therefore crucial in laboratory medicine practice. Well-known general references^3,5–9^ and a case for teaching tolerance intervals in introductory statistics courses^10^ are available.

It is common practice to assume that clinical markers related to a disease follow a normal distribution among healthy subjects. If there is evidence against this assumption we could fit models to specify optimal transformations to normality, e.g. logarithmic or square root though this might still result in biased estimates of the upper or lower limits of the reference range depending on whether the distribution is right or left skewed.^9^ Alternatively we could construct reference ranges under specific parametric assumptions different to normality, or follow a nonparametric procedure. The focus of this paper is on the construction of parametric and nonparametric reference ranges for a selected reference population based on a random sample from the population. The problems related to selecting a reference population have been discussed elsewhere.^6^

A *P* (commonly set to 95%) reference range is a data-based interval that purports to include (100 P)% of the values in the population of interest. Their main point is to classify any future observations from the population which fall outside these intervals as atypical and thus may warrant further investigation.

Let F(·) denote the continuous cumulative distribution function (cdf) of the population, and $F^{-1}(\gamma )$ denote the (100 γ)-th percentile of the population for a given γ∈(0,1). The interval $\left[F^{-1}\left((1-P)/2\right),F^{-1}\left((1+P)/2\right)\right]$ contains exactly (100 P)% of the population and would be used as the *P* reference range had *F* been known. Since F(·) is usually not known completely in real problems, the reference range has to be estimated from a random sample $X_{1},\ldots ,X_{n}$ from the population, i.e. $X_{1}\ldots ,X_{n}$ are independent random variables identically distributed F(·). Note that we follow the notation in Krishnamoorthy and Mathew^11^ thus denoting the interval’s content level by *P* instead of the commonly used 1−α, and its confidence level by *γ*.

When F(·) is assumed to have a normal distribution $N(\mu ,\sigma^{2})$ with unknown mean *μ* and unknown variance $\sigma^{2}$, we have $F^{-1}(\gamma )=\mu +z_{\gamma }\;\sigma$ where $z_{\gamma }$ denotes the (100 γ)-th percentile of the standard normal distribution *N*(0, 1). When F(·) is not assumed to have a parametric form, nonparametric (or distribution free) methods can be used. In this paper, both normal-based and nonparametric methods are considered.

As a reference range depends on the random sample, the proportion of the population contained in it is also random. Thus the question is ‘which statistical intervals should be used as reference ranges?’

In this article we argue that a *P* prediction interval, which continues to be used as a reference range in the literature,^6,12,13^ is not fit for the purpose of interest since there is a substantial probability (due to the randomness in the sample) that the prediction interval contains less than (100 P)% of the population.

We then argue that a (P,γ) tolerance interval, with confidence γ∈(0,1) set at a pre-specified large value, γ=0.95 say, is valid as a reference range since it guarantees, with large confidence *γ* due to the randomness in the sample, to contain (100 P)% of the population values. Several authors have proposed to use tolerance intervals as reference ranges.^5,14,15^ With almost 80 years of research on tolerance intervals or regions, various parametric and nonparametric procedures are readily available for use as reference ranges.

The next two sections discuss reference ranges based on the normal distribution, and nonparametric reference ranges. They are followed by a section considering a numerical example, and a final one with concluding remarks.

## 2 Reference ranges based on the normal distribution

### 2.1 Reference ranges currently in use

Based on the sample, one reference range that has been widely used is the *P* prediction interval for a future observation *Y* from a population with $N(\mu ,\sigma^{2})$ distribution^6,12,13^

$$RR_{1}=\bar{X}\pm t_{(1+P)/2,\nu }\;S\sqrt{1+1/n}=\bar{X}\pm c_{1}\;S$$

where $\bar{X}=\frac{1}{n}\sum_{i=1}^{n}X_{i}$ is the sample mean, $S^{2}=\frac{1}{n-1}\sum_{i=1}^{n}{\left(X_{i}-\bar{X}\right)}^{2}$ is the sample variance, $t_{\delta ,\nu }$ is the (100 δ)-th percentile of the *t* distribution with *ν* degrees of freedom (df), ν=n−1, and $c_{1}=t_{(1+P)/2,\nu }\sqrt{1+1/n}$.

A relevant guide on prediction intervals for reference regions is available,^7^ and we note that the prediction interval *RR*_1_ has also been called the *P* expectation tolerance interval.^16,17^

Other reference ranges are based on estimators of the percentiles $\mu \pm z_{(1+P)/2}\;\sigma$ and include

$$\begin{array}{l} RR_{2}=\bar{X}\pm z_{(1+P)/2}\;S=\bar{X}\pm c_{2}\;S\\ RR_{3}=\bar{X}\pm z_{(1+P)/2}\;S/\lambda_{\nu }=\bar{X}\pm c_{3}\;S\\ RR_{4}=\bar{X}\pm z_{(1+P)/2}\;S\;\lambda_{\nu }=\bar{X}\pm c_{4}\;S \end{array}$$

where $\lambda_{\nu }=\sqrt{2/\nu }\;\Gamma \;\left((\nu +1)/2\right)\;/\;\Gamma (\nu /2),\;c_{2}=z_{(1+P)/2}$, $c_{3}=z_{(1+P)/2}\;/\;\lambda_{\nu }$ and $c_{4}=z_{(1+P)/2}\;\lambda_{\nu }$.^9,12^ Now $\bar{X}+c_{2}\;S$ is a naïve estimator of $\mu +z_{\gamma }\;\sigma ,\;\bar{X}+c_{3}\;S$ has the minimum variance among unbiased estimators of $\mu +z_{\gamma }\sigma$, and $\bar{X}+c_{4}\;S$ has minimum mean squared error among estimators of the form $\bar{X}+c\;S$ where *c* is a constant.^12^

One immediate question is whether these reference ranges *RR_i_* contain (100 P)% of the values in the population, which is the objective of a reference range. Note that the proportion of the population within the reference range $RR_{i}=\bar{X}\pm c_{i}\;S$ is given by

(1) $$K_{i}=\underset{Y\;|\;X_{1},\ldots ,X_{n}}{\mathrm{Pr}}\left\{Y\in \bar{X}\pm c_{i}S\right\}=\Phi \left(\frac{\bar{X}-\mu }{\sigma }+c_{i}\frac{S}{\sigma }\right)-\Phi \left(\frac{\bar{X}-\mu }{\sigma }-c_{i}\frac{S}{\sigma }\right)$$

where $Y∼N(\mu ,\sigma^{2})$ and is independent of the sample $X_{1},\ldots ,X_{n}$, $\underset{Y\;|\;X_{1},\ldots ,X_{n}}{\mathrm{Pr}}\{\cdot \}$ is the conditional probability of *Y* conditioning on the sample $X_{1},\ldots ,X_{n}$, and Φ(·) is the cdf of a *N*(0, 1) random variable. Hence the objective of a reference range is to have $K_{i}\geq P$. It is clear from equation (1) that *K_i_* is a random variable depending on the random sample via $\bar{X}$ and *S* so whether ‘$K_{i}\geq P$’ is also random. As a result, all we can hope is that the event $\left\{K_{i}\geq P\right\}$ has a large probability of occurrence.

**Figure 1.**
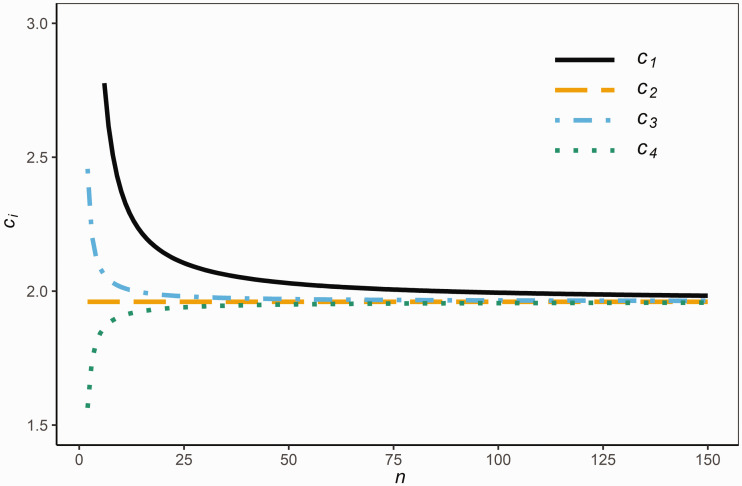
The value of *c_i_* as a function of the sample size *n.*

We note from equation (1) that *K_i_* increases as *c_i_* increases. Hence, among the *RR_i_* (1≤i≤4) given above, the one that has the largest *c_i_* contains the largest proportion of the population. Figure 1 compares the *c_i_* for given sample sizes n=2:150 and *P *=* *0.95. Clearly, *c*_1_ is the largest among the *c_i_* (1≤i≤4), and so *RR*_1_ contains the largest proportion of the population among the four reference ranges. We therefore investigate whether or not ‘$K_{1}\geq P$’ has a large probability to occur in order for *RR*_1_ to be used as a reference range.

First, note that

(2) $$\text{E}\;(K_{1})=\text{E}\left\{\underset{Y\;|\;X_{1},\ldots ,X_{n}}{\mathrm{Pr}}\left\{Y\in \bar{X}\pm c_{1}S\right\}\right\}=\mathrm{Pr}\left\{Y\in \bar{X}\pm c_{1}S\right\}$$

(3) $$\;\;\;=\mathrm{Pr}\left\{\frac{\left|Y-\bar{X}\right|/\left(\sigma \sqrt{1+1/n}\right)}{S/\sigma }\;<\;t_{P/2,\nu }\right\}=P$$

where the equality in equation (2) results directly from the well-known conditional expectation formula,^18^ and the equality in equation (3) follows from the fact that $\left(Y-\bar{X}\right)/\left(\sigma \sqrt{1+1/n}\right)$ is distributed *N*(0, 1) and is independent of S/σ which has the distribution $\sqrt{\chi_{\nu }^{2}/\nu }$, with $\chi_{\nu }^{2}$ denoting a chi-squared random variable with ν=n−1 df. That the probability in equation (3) is equal to *P* qualifies *RR*_1_ as a *P* prediction interval for a future observation *Y* from the same population that the sample $X_{1},\ldots ,X_{n}$ is drawn.

**Figure 2.**
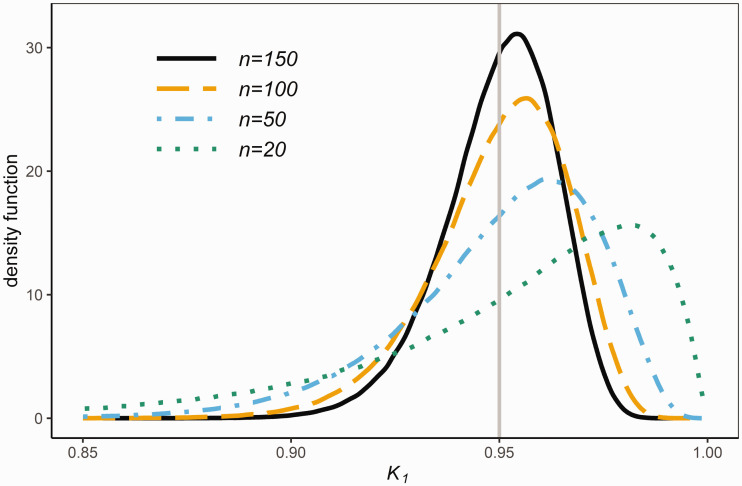
The pdf’s of *K*_1_ for various sample sizes *n.*

Second, the distribution of *K*_1_ can be studied by simulating a large number, $R_{\mathrm{sim}}=1,000,000$ say, of independent realisations of *K*_1_. Note from equation (1) that

(4) $$K_{1}=\Phi \left(\frac{Z}{\sqrt{n}}+c_{1}\;\sqrt{\frac{\chi_{\nu }^{2}}{\nu }}\right)-\Phi \left(\frac{Z}{\sqrt{n}}-c_{1}\;\sqrt{\frac{\chi_{\nu }^{2}}{\nu }}\right)$$

where $Z=\sqrt{n}(\bar{X}-\mu )/\sigma$ is a standard *N*(0, 1) random variable, $\chi_{\nu }^{2}=\nu \;S^{2}/\sigma^{2}$ is a chi-squared random variable with ν=n−1 df, and *Z* and $\chi_{\nu }^{2}$ are statistically independent. From equation (4), *K*_1_ can easily be simulated. For given *P* and *n*, $R_{\mathrm{sim}}=1,000,000$ replicas of *K*_1_ are simulated, based on which the probability density function (pdf) of *K*_1_ can be accurately approximated. In Figure 2, the kernel density estimate^19^ of the pdf of *K*_1_ based on the simulated *K*_1_ values is plotted (by using the R package KernSmooth)^20^ for n=20, 50, 100 and 150. Based on the simulated $K_{1}$ values, we approximated $\mathrm{Pr}\{K_{1}<P\}$ by the proportion of the $K_{1}$ values that are less than P=0.95, which are given by 0.385, 0.429, 0.450 and 0.459 for n=20, 50, 100 and 150, respectively. Note that $\mathrm{Pr}\{K_{1}<P\}$ is given in Figure 2 by the area under the pdf to the left of the vertical line at P=0.95.

Given equation (3), it can be shown by the delta method that $\sqrt{n}\;\left(K_{1}-P\right)$ tends when n→∞ to a normal distribution with zero mean and finite variance. This is supported by Figure 2 which shows that the pdf of *K*_1_ is getting closer to be symmetric and centered with decreasing variance at *P* as *n* increases. Note that *n *=* *150 is not large enough yet for the pdf of *K*_1_ to converge to a normal pdf. From a brief simulation study we found that in order to achieve this satisfactorily the sample size must be very large indeed. Even for *n *=* *10, 000 the skewness and kurtosis values suggest a significant lack of normality. The coefficient of variation of *K*_1_ for *n *=* *150 is 0.014, and becomes smaller than 0.01 for n≥300, and is around 0.002 for *n *=* *10,000. This asymptotic normal distribution implies that $\mathrm{Pr}\{K_{1}<P\}\to 0.5$ as n→∞, that is, the probability of the reference range *RR*_1_ containing less than (100 P)% of the population is about 1/2 when the sample size is large.

The argument above means that, due to the sample’s randomness, using *RR*_1_ as the reference range implies that there is a substantive probability, close to 50% when *n* is sufficiently large, that the reference range does not fulfill its objective of containing (100 P)% of the population. Its property $\text{E}\;(K_{1})=P$ in equation (3) has the following interpretation. A large number of individuals, *I* say, collect independent samples, one each, and compute the corresponding reference ranges *RR*_1_ based on their own samples. Then the proportions of the population contained in these *I* reference ranges, $K_{1,1},\ldots ,K_{1,I}$, are random values from the interval (0, 1) and form a random sample from the distribution of *K*_1_ although some values could be very close to 0 and some values could be very close to 1. The property $\text{E}\;(K_{1})=P$ merely says that $(K_{1,1}+\cdots +K_{1,I})/I$ is close to *P* when *I* is large. Hence, the proportion of the population that one particular reference range contains could be very small but this is compensated by some very large proportions of the population that some other individuals’ reference ranges might contain in the sense that $(K_{1,1}+\cdots +K_{1,I})/I$ is close to *P*. This potential for compensation from other reference ranges is unlikely to offer any comfort for knowing that one’s reference range has a substantial probability of containing less than (100 P)% of the population. It is clearly desirable to have a high confidence that our own reference range contains (100 P)% of the population. Hence *RR*_1_ falls short on this ground and should not be used as a reference range.

The justification for using prediction intervals as reference ranges^5,13^ is that exactly (100 P)% of the future observations from the population should fall within the prediction intervals. It is clear from the line of reasoning stated in the previous paragraphs that this argument is not valid. The inappropriateness of prediction regions when used as reference regions has also been noted in Sections 2.2 and 3.3 of Dong and Mathew.^15^

In the next section we discuss tolerance intervals since several authors^5,14,15^ have proposed to use them as reference ranges. For example it has been stated that ‘it would seem that the statistical tolerance interval is what clinical chemists have in mind when they speak of a reference range derived from a sample of individuals representing some defined population’^5^ (p. 55).

### 2.2 Tolerance intervals

A tolerance interval with content level *P* is a data-based random interval constructed to contain (100 P)% of the population with a pre-specified (large) confidence level *γ* about the randomness in the sample.^11,16,17,21–23^ Specifically, a (P, γ) tolerance interval is given by^11^

$$RR_{5}=\bar{X}\pm c_{5}\;S$$

where the critical constant $c_{5}=c_{5}\;(P,\gamma ,n)$ is chosen such that

(5) $$\begin{array}{l} \mathrm{Pr}\left\{\underset{Y\;|\;X_{1},\ldots ,X_{n}}{\mathrm{Pr}}\left\{Y\in \bar{X}\pm c_{5}S\right\}\geq P\right\}=\mathrm{Pr}\left\{\Phi \left(\frac{\bar{X}-\mu }{\sigma }+c_{5}\frac{S}{\sigma }\right)-\Phi \left(\frac{\bar{X}-\mu }{\sigma }-c_{5}\frac{S}{\sigma }\right)\;\geq P\right\}\\ =\mathrm{Pr}\left\{\Phi \left(Z/\sqrt{n}+c_{5}\sqrt{\chi_{\nu }^{2}/\nu }\right)-\Phi \left(Z/\sqrt{n}-c_{5}\sqrt{\chi_{\nu }^{2}/\nu }\right)\;\geq P\right\}=\gamma \end{array}$$

where the random variables *Z* and $\chi_{\nu }^{2}$ in equation (5) are the same as those in equation (4). The R package tolerance^24,25^ can be used to compute *c*_5_.

**Figure 3.**
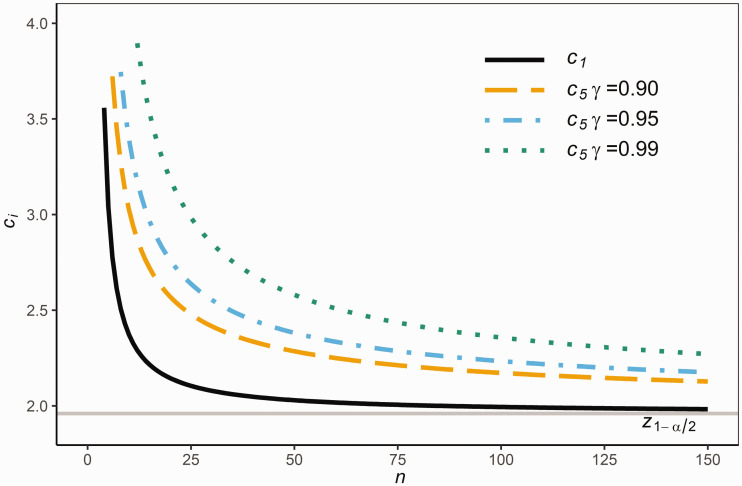
The values of *c*_1_ and *c*_5_ for various sample sizes *n.*

Figure 3 compares *c*_1_ and *c*_5_ for given sample sizes n=2:150 with *P *=* *0.95 and γ={0.90,0.95}. It is clear from Figure 3 that *c*_5_ is considerably larger than *c*_1_ in order that *RR*_5_ contains (100 P)% of the population with a pre-specified large confidence *γ* about the randomness in the sample. Also, as expected, *c*_5_ increases with *γ* as seen in Figure 3.

## 3 Equal-tailed tolerance intervals

The tolerance interval *RR*_5_ contains (100 P)% of the population with a pre-specified (large) confidence *γ* about the randomness in the sample. But the proportion *P* of the population contained in *RR*_5_ may not be the central (100 P)% interval of the population. If we insist that a reference range should contain that central proportion of the population, i.e. $\left[\mu -z_{(1+P)/2}\;\sigma ,\;\mu +z_{(1+P)/2}\;\sigma \right]$ with pre-specified confidence *γ* about the randomness in the sample, then we should use the following interval as the reference range

$$RR_{6}=\bar{X}\pm c_{6}\;S$$

where the critical constant $c_{6}=c_{6}\;(P,\gamma ,n)$ is chosen such that

$$\mathrm{Pr}\left\{\bar{X}-c_{6}\;S<\mu -z_{(1+P)/2}\sigma \;\;\text{ and }\;\;\mu +z_{(1+P)/2}\sigma <\bar{X}+c_{6}\;S\right\}=\gamma \;$$

This interval is called the equal-tailed or central (P,γ) tolerance interval.^15^ A formula for values of *c*_6_ is available^11^ and can be computed using the function K.factor of the R package tolerance.^24,25^ This interval can be viewed as a *γ* confidence simultaneous lower confidence bound on quantile $\mu -z_{(1+P)/2}\;\sigma$ and upper confidence bound on quantile $\mu +z_{(1+P)/2}\;\sigma$.^26^

It is clear that comprising the central (100 P)% of the population $\left[\mu -z_{(1+P)/2}\;\sigma ,\;\mu +z_{(1+P)/2}\;\sigma \right]$ implies containing (100 P)% of the population. Hence the equal-tailed *RR*_6_ satisfies a more stringent requirement than *RR*_5_ and, as a result, *c*_6_ is larger than *c*_5_.

**Figure 4.**
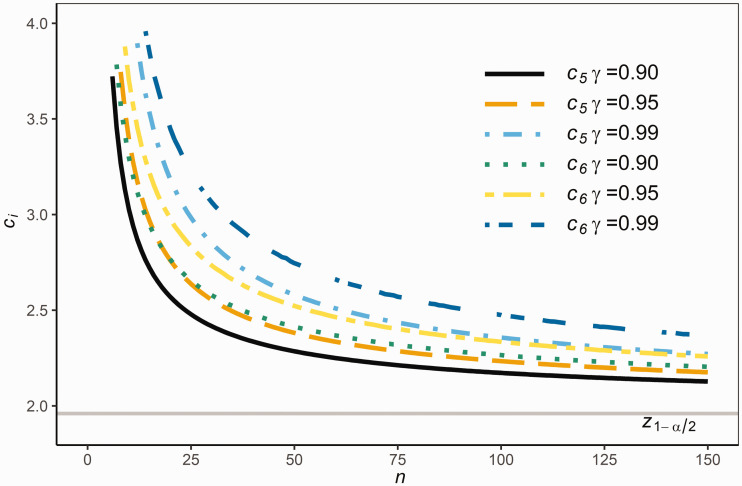
The values of *c*_5_ and *c*_6_ for various sample sizes *n.*

Figure 4 compares *c*_5_ and *c*_6_ for given sample sizes n=2:150 with *P *=* *0.95 and confidence γ={0.90,0.95,0.99}. It is clear from Figure 4 that $c_{6}>c_{5}$, as expected.

Our view is that the (P,γ) tolerance interval should be used as the reference range since its form $\bar{X}\pm c_{5}\;S$ is centered at $\bar{X}$, mimicking the form of the equal-tailed tolerance interval $\mu \pm c_{6}\;\sigma$, and with a large confidence *γ* it does contain (100 P)% of the population. Only if we specifically require the reference range to contain the central (100 P)% of the population, $\mu \pm z_{(1+P)/2}\sigma$, then the equal-tailed (P,γ) tolerance interval should be used; otherwise it is unnecessarily wider and flags as atypical fewer individuals than the (P,γ) tolerance interval.

## 4 Nonparametric reference ranges

### 4.1 Nonparametric prediction intervals

When F(·) is not assumed to have a specific form, nonparametric reference ranges can be considered and are based on the order statistics $X_{\left[1\right]}<\ldots <X_{\left[n\right]}$ of the sample $X_{1},\ldots ,X_{n}$, and the sample quantiles have been used to estimate the population quantiles $F^{-1}\left(\left(1-P\right)/2\right)$ and $F^{-1}\left(1+P)/2\right)$.^6^

In what follows, $j^{\left(p\right)}$ and $j^{\left(t\right)}$ are indices used for prediction and tolerance intervals, respectively.

Let $j^{\left(p\right)}$, with $1\leq j^{\left(p\right)}\leq n/2$, be the largest natural number such that

(6) $$\mathrm{Pr}\left\{Y\in (X_{\left[j^{\left(p\right)}\right]},X_{\left[n-j^{\left(p\right)}+1\right]})\right\}\geq P\;$$

where *Y* is a future observation from the population F(·) independent of the random sample $X_{1},\ldots ,X_{n}$ as before. Using the well-known facts that $U_{1}=F(X_{1}),\ldots ,U_{n}=F(X_{n})$ are independent, each having a uniform distribution on the interval (0, 1), and that $U_{\left[k\right]}=F(X_{\left[k\right]})$ is the *k*-th order statistic of $U_{1},\ldots ,U_{n}$ and has a beta distribution with parameters *k* and n−k+1, the probability in (6) is equal^16^ to $\left(n+1-2\;j^{\left(p\right)}\right)/(n+1)$. Hence the constraint on $j^{\left(p\right)}$ required in equation (6) gives

(7) $$j^{\left(p\right)}=〈(n+1)\;\left(1-P\right)/2〉$$

where 〈a〉 denotes the integer part of *a*. This leads to use the nonparametric prediction interval

$$RR_{7}=\left(X_{\left[j^{\left(p\right)}\right]},X_{\left[n-j^{\left(p\right)}+1\right]}\right)$$

as a reference range. An interesting remark is that $X_{\left[j^{\left(p\right)}\right]}$ and $X_{\left[n-j^{\left(p\right)}+1\right]}$ are consistent point estimators of the population quantiles $F^{-1}\left((1-P)/2\right)$ and $F^{-1}\left((1+P)/2\right)$, respectively.

The proportion of the population contained in *RR*_7_ is given by

(8) $$\begin{array}{l} K_{7}=\underset{Y\;|\;X_{1},\ldots ,X_{n}}{\mathrm{Pr}}\left\{Y\in (X_{\left[j^{\left(p\right)}\right]},X_{\left[n-j^{\left(p\right)}+1\right]})\right\}\;\\ =\underset{Y\;|\;X_{1},\ldots ,X_{n}}{\mathrm{Pr}}\left\{F(Y)\in \left(F(X_{\left[j^{\left(p\right)}\right]}),F(X_{\left[n-j^{\left(p\right)}+1\right]})\right)\right\}\;\\ =U_{\left[n-j^{\left(p\right)}+1\right]}-U_{\left[j^{\left(p\right)}\right]} \end{array}$$

which is a random variable. The important question is whether the probability that this proportion is at least *P*, given by

(9) $$\mathrm{Pr}\left\{U_{\left[n-j^{\left(p\right)}+1\right]}-U_{\left[j^{\left(p\right)}\right]}\geq P\right\}\;$$

is sufficiently large to qualify the *P* prediction interval $RR_{7}=\left(X_{\left[j^{\left(p\right)}\right]},X_{\left[n-j^{\left(p\right)}+1\right]}\right)$ as a reference range.

By noting that $U_{\left[n-j^{\left(p\right)}+1\right]}-U_{\left[j^{\left(p\right)}\right]}$ and $U_{\left[n-2j^{\left(p\right)}+1\right]}$ follow the same beta distribution $B_{n-2j^{\left(p\right)}+1,2\;j^{\left(p\right)}}$, Tukey’s equivalence blocks result^27^ directly implies that

(10) $$\mathrm{Pr}\left\{U_{\left[n-j^{\left(p\right)}+1\right]}-U_{\left[j^{\left(p\right)}\right]}\geq P\right\}\;=\;1-B_{n-2j^{\left(p\right)}+1,2j^{\left(p\right)}}(P)$$

where $B_{n-2\;j^{\left(p\right)}+1,2j^{\left(p\right)}}(\cdot )$ denotes the cdf of the beta distribution with parameters $n-2\;j^{\left(p\right)}+1$ and $2\;j^{\left(p\right)}$. This probability can be easily calculated using the function pbeta in R.

Note that, as n→∞, the beta distribution $B_{n-2\;j^{\left(p\right)}+1,2\;j^{\left(p\right)}}$ converges to a normal distribution with mean *P* thus the probability in equation (10) approaches 0.5 as n→∞.

**Figure 5.**
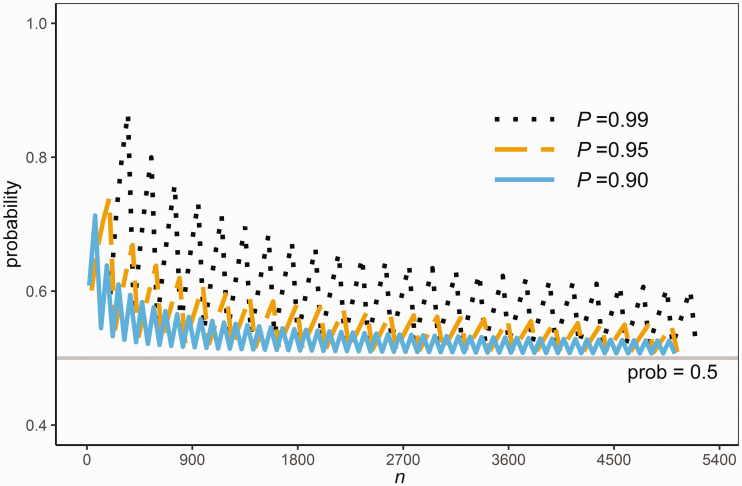
The probability in equation (10) for various sample sizes *n.*

Figure 5 plots this probability against *n* for P={0.90,0.95,0.99}. The plots are saw-tooth shaped due to the discreetness of *n* and $j^{\left(p\right)}$. It is clear from the figure that this probability can be substantially smaller than *P*, and approaches 0.5 as *n* is large as expected from the asymptotic normal distribution pointed out above. This shows that the nonparametric prediction interval has a substantial probability, close to 0.5 when *n* is large, of containing less than (100 P)% of the population values. Hence, this nonparametric prediction interval should not be used as a reference range for the same reason as the prediction interval based on the normal distribution.

## 5 Nonparametric tolerance intervals

A nonparametric tolerance interval is constructed to contain (100 P)% of the population with a pre-specified (large) confidence *γ* about the randomness in the sample. Consider the following nonparametric tolerance interval^21^

$$RR_{8}=(X_{\left[j^{\left(t\right)}\right]},\;X_{\left[n-j^{\left(t\right)}+1\right]})$$

where $j^{\left(t\right)}$ satisfies that $1\leq j^{\left(t\right)}\leq n/2$ should be the largest natural number such that the proportion of the population contained in *RR*_8_, given by

$$K_{8}=\underset{Y\;|\;X_{1},\ldots ,X_{n}}{\mathrm{Pr}}\left\{Y\in (X_{\left[j^{\left(t\right)}\right]},X_{\left[n-j^{\left(t\right)}+1\right]})\right\}\;=\;U_{\left[n-j^{\left(t\right)}+1\right]}-U_{\left[j^{\left(t\right)}\right]}$$

following similar lines as *K*_7_ in equation (8), is at least *P* with probability *γ* about the randomness in the sample $X_{1},\ldots ,X_{n}$. It follows therefore from equation (10) that $1\leq j^{\left(t\right)}\leq n/2$ should be the largest natural number that satisfies

(11) $$\mathrm{Pr}\left\{U_{\left[n-j^{\left(t\right)}+1\right]}-U_{\left[j^{\left(t\right)}\right]}\geq P\right\}\;=\;1-B_{n-2j^{\left(t\right)}+1,2j^{\left(t\right)}}(P)\;\geq \;\gamma$$

For given *n*, *P* and *γ*, $j^{\left(t\right)}$ can be easily computed by a direct search over the natural numbers in the range from 1 to n/2. Note that if the sample size *n* is too small, then the existence of $j^{\left(t\right)}$ is not guaranteed unless *n* satisfies^11^

(12) $$1-\left(n\;P^{n-1}-(n-1)\;P^{n}\right)\geq \gamma$$

The equal-tailed or central nonparametric tolerance intervals can be constructed in a similar way. Our view is that a (P,γ) nonparametric tolerance interval is pertinent as a reference range similar to the normal distribution case. Hence we do not go into the details about the equal-tailed nonparametric tolerance intervals to save space.

**Figure 6.**
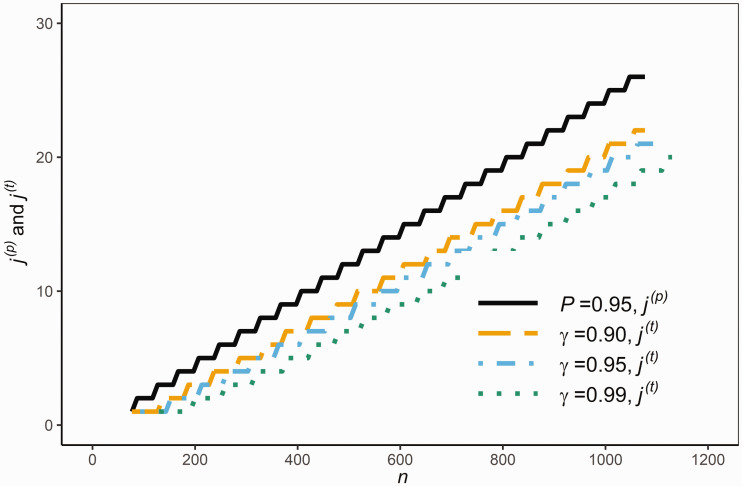
The values of $j^{\left(p\right)}$ and $j^{\left(t\right)}$ for various sample sizes *n* given *P* and *γ.*

Figure 6 compares $j^{\left(t\right)}$ and $j^{\left(p\right)}$ for given sample sizes *n* with *P *=* *0.95 and γ={0.90,0.95,0.99}. It is clear from Figure 6 that $j^{\left(t\right)}$ is considerably smaller than $j^{\left(p\right)}$, and so *RR*_8_ is wider than *RR*_7_, in order that *RR*_8_ contains (100 P)% of the population with a pre-specified large confidence *γ* about the randomness in the sample. Also, as expected, $j^{\left(t\right)}$ decreases as *γ* increases.

## 6 Example

A random sample of *n *=* *210 observations on fasting plasma glucose is taken from the population of interest. The data and the R code for all the computations in this paper are available at http://www.personal.soton.ac.uk/wl/RefRange/.

Suppose that the usual normality tests^28^ show that it is reasonable to assume the population has a normal distribution. The sample mean and standard deviation are computed to be $\bar{X}=5.31$ and *S *=* *0.41 (in unit mmol/L). If we use the prediction interval as the reference range, then it is given by

$$\begin{array}{l} RR_{1}=\bar{X}\pm t_{(1+P)/2,\nu }\;S\;\sqrt{1+1/n}=5.31\pm 1.97\;\times \;0.41\;\times \;\sqrt{1+1/210}\\ =[4.49,\;6.12] \end{array}$$

Note, however, as pointed out above, that the probability of the prediction interval containing less than (100 P)% of the population can be substantial and is computed to be 47%. So there is a 47% probability that the interval does not do what it purports to do: containing (100 P)% of the population.

If we use the (P,γ) tolerance interval as the reference range, with γ=0.95, then it is given by

$$RR_{5}=\bar{X}\pm c_{5}\;S=5.31\pm 2.14\;\times \;0.41=[4.43,\;6.19]$$

This interval is wider than the prediction interval. But, as we pointed out, the tolerance interval does contain (100 P)% of the population with probability γ=0.95. Therefore, any future observations falling outside this interval can be regarded as atypical and should be considered for further investigation.

While the tolerance interval above has a confidence γ=95% of containing (100 P)% of the population, it has a less than γ=95% probability of containing the central (100 P)% of the population, $\mu \pm z_{(1+P)/2}\sigma$. This probability is computed to be 86%.

In order to have a γ=95% probability of containing the central (100 P)% of the population, $\mu \pm z_{(1+P)/2}\sigma$, we can use the equal-tailed (P,γ) tolerance interval, which is given by

$$RR_{6}=\bar{X}\pm c_{6}\;S=5.31\pm 2.21\;\times \;0.41=[4.40,\;6.22]$$

The confidence that this equal-tailed tolerance interval contains (100 P)% of the population is computed to be 99%, which is much larger than γ=95%. Hence, with a 99% probability, the equal-tailed tolerance interval contains (100 P)% of the population. Furthermore, we estimated that the equal-tailed tolerance interval $\bar{X}\pm 2.21\;S$ is the (0.957,γ) tolerance interval, that is, the interval contains 95.7% of the population with confidence γ=95%.

Now suppose that the distribution of the population cannot be assumed to be normal. Then nonparametric reference ranges should be used. If we use the prediction interval as the reference range, then it is given by

$$RR_{7}=[X_{\left[5\right]},\;X_{\left[n-5+1\right]}]=[X_{\left[5\right]},\;X_{\left[206\right]}]=[4.62,\;6.09]$$

with $j^{\left(p\right)}=5$. Note, however, as we have pointed out, that the probability of the prediction interval containing less than (100 P)% of the population can be substantial and is computed to be 39%. So there is a 39% probability that the interval does not do what it purports to do: containing (100 P)% of the population.

If we use the (P,γ) nonparametric tolerance interval as the reference range, with γ=0.95, then it is given by

$$RR_{8}=[X_{\left[3\right]},\;X_{\left[n-3+1\right]}]=[X_{\left[3\right]},\;X_{\left[208\right]}]=[4.38,\;6.27]$$

with $j^{\left(t\right)}=3$. This tolerance interval is wider than the nonparametric prediction interval but, as we pointed out, it does contain (100 P)% of the population with 95% confidence. Therefore, any future observations falling outside this interval can be regarded as atypical and should be considered for further investigation.

Finally, we note that nonparametric intervals are usually wider than the corresponding parametric ones since they require fewer assumptions than the parametric model.

## 7 Conclusions

The objective of a reference range is to contain a pre-specified large content level (100 P)% of the population with *γ* confidence level, so that a future observation falling outside the reference range is regarded as atypical and considered for further investigation. This procedure should be useful as part of screening programmes, whose aim is to identify subjects at sufficient risk of a specific disorder who may benefit from further investigation or direct preventive action to avoid death or disability and to improve their quality of life.^29^

Since a reference range depends on the random sample, the event ‘a reference range contains (100 P)% of the population’ is also random and so we can never be certain that a reference range contains (100 P)% of the population. All we can hope for is that the event ‘a reference range contains (100 P)% of the population’ occurs with a large probability, *γ*.

Based on this premise, we have argued that the prediction interval is not suitable as a reference range since there is a substantial probability, close to 50% when *n* is large, that the prediction interval contains less than (100 P)% of the population. In contrast, a (P,γ) tolerance interval is designed to contain (100 P)% of the population with a pre-specified large confidence *γ* so it is eminently adequate as a reference range.

Tolerance intervals or regions have been studied by many statisticians since the 1940s. Various parametric and nonparametric procedures are readily available for use as reference ranges or reference regions.^11,16,17,24^ Finally, we note that there is some work on constructing reference ranges specifically assuming that the clinical marker follows a log-normal distribution,^30^ and on sample size calculation for reference ranges,^31,32^ and tolerance intervals.^33–35^ These aspects, however interesting, fall beyond the scope of our paper.
